# Extracts from Discussions of the Mississippi Valley Association of Dental Surgeons

**Published:** 1867-05

**Authors:** 


### Proceedings of Societies.

---

### EXTRACTS FROM DISCUSSIONS OF THE MISSISSIPPI VALLEY ASSOCIATION OF DENTAL SURGEONS.

BY ITS OWN REPORTER.

### MECHANICAL DENTISTRY.

Dr. Shadoan said with regard to impressions, he remembered seeing it stated a good while ago, that you might smoke a plaster impression with any resinous substance—make it black with smoke.

A few months ago he concluded to try gas, which answered the purpose quite well. A coal oil lamp or candle would do as well. It left no spaces or interstices, and could easily be separated in a few minutes. It was nothing new, but he had forgotten it.

Dr. C. M. Kelsey knew there was danger of varnish or oil getting into the interstices, and it was a question in his mind if the amount of soot that might be deposited would not make this mode objectionable.

Dr. Shadoan inquired if any one had hardened casts in water. He had seen plaster casts treated thus. Just as soon as the plaster was set sufficiently, it was plunged into water, and let remain two or three days, when it came out very hard indeed.

Dr. Berry simply desires to ask a question. In grinding teeth up he found it difficult sometimes to make a close, nice joint. He had seen some in the faculty room of the college that had such nice joints. He would like to know how they were ground, and if it can be done on an ordinary grinder?

The specimens referred to were exhibited to the association, and Dr. S. S. White, of Philadelphia, remarked that they were some that had been ground up by a young man in his employ, who has been a regular mechanical Dentist. It required nothing more than care with a good corundum wheel.

It might be proper to state, since they had been kind enough to ask about them, that he did not bring them to exhibit them before this body. They were among some that were prepared for the Paris exhibition, but were not the best he had, and he brought them along to show some friends in Chicago. He did not intend to intrude them on this society at all.

### NEW APPLIANCES.

Automatic Plugger—exhibited and described.

This instrument was exhibited a year ago. It acts more directly upon the system than any other automatic plugger. The strength of the blow may be regulated by a contrivance at the top.

If the screw is put down it gives a harder blow, and by raising it the strength of the blow is diminished.

It rotates so as to give any position the operator wishes in filling teeth.

Gas Generator—exhibited and described by Dr. Ulrey, who said that it would melt lead in three-quarters of a minute. The air contained in the vessel would last half a day, would take about a half gallon benzine, worth 15 cents to last a day in the use of it. Had used it for twelve months and found it the best thing for vulcanizing he ever used. The heat could be raised to 280° in from five to seven minutes.

Gold could be melted over the fire, water boiled, and meat roasted or boiled as well as over any fire.

The tube or pipe could be put on whenever needed, and used for soldering purposes. For silversmiths and jewelers it is as good a thing for soldering as to be found anywhere.

The force was produced by compressed atmosphere, the atmosphere was compressed to four or five times less than its original bulk, by a few strokes of the force pump attached. Is entirely free from smoke for any length of time. With coal oil as generally used there was more or less smoke. The fire is easily regulated, if the thermometer indicated that the heat was too great.

There was no danger of explosion, had tried it in every way he could think of. His was not as good as the one on exhibition, but was one of the first made.

The cheapness was what he looked at; the price being but $10. They are made of various sizes and shapes to suit the fancy of different persons. They were originated and are manufactured by Mr. John S. Hall, 61 Milton street, Cincinnati.

Ether Spray Producer, exhibited and described by Mr. Hall, who said that the instrument had only been gotten up on yesterday by a gentleman, at his suggestion; and because so hurriedly made was a crude affair to what it might have been, had there been more time for its construction.

The maker proposed to make them of any size or shape desired. The price would be from $8 to $10. They were much more convenient than the india rubber bag.

The operation of the machine was fully shown, the Doctor throwing a fine stream of spray from it to any distance or part of the room he desired.

The combined Lip and Cheek Holder, was next exhibited and described by Dr. C. Bradley, of Dayton. The manner in which this appliance is used was shown by using the president, Dr. Goddard, as a patient. The belt which comprised a portion of the invention, was first adjusted so as to raise the upper lip of the subject, giving the operator a fair chance at the upper teeth, using the small plugger with the right hand, and the mallet with the left hand.

A small flat silver hook was then attached to the belt, and hooked in the side of the mouth, and the belt drawn around the side of the patient's face and back of the head, over the patient's shoulder; the end of the belt in each instance being held by the patient's own hand. A small napkin folded up was placed on the side of the cheek near the hook, which answered the purpose of a fulcrum. In this way the cheek was drawn away from the side teeth. Another portion of the appliance consisted of a little silver attachment, called the "extension" which by being fastened to the end of the hook extended back inside of the mouth, and by the use of a small folded napkin being placed under it, prevented the flow of saliva in the mouth.

The exhibitor remarked that this appliance was gotten up to dispense with an assistant; it left both hands of the operator free: he would not have an assistant; could use this to much better advantage than an assistant; he knew just where to strike, how hard to strike and all about it. The habit of using the mallet in the left hand was very easily acquired, much easier than would be generally supposed. He would supply them at $15 each. As the metal portions were made of pure silver, he did not derive much profit from their manufacture.

A vote of thanks was tendered Dr. Bradley for his invention, and the manner in which he explained its use.

Dr. C. W. Spalding remarked, that the exhibition of this invention was worth all the discussions and exhibitions he had seen for years.

Dr. Geo. Watt said, he could bear testimony to the value of the instrument, having seen it tested a few times; and it made him almost sick that he did not have it years ago to enjoy, as he did not fill teeth now.

A Piston Plugger was exhibited by Dr. G. W. Keely, quite inferior to the improved ones made now, and was somewhat out of order, so that he could not show the manner in which it worked as he would like. It was invented by Dr. Poor, of Dubuque, Iowa. He first saw it used in the hands of Dr. Scroggs, of Galena, who was generally acknowledged as a fine operator. When he first showed the way in which it was used, remarked Dr. Keely, "I was never more captivated than with it, and felt there was more brain in it than any thing the profession had in the way of pluggers. By means of a small spiral screw inside, the force of the blow was given while the point of the instrument against the tooth remains stationary.

Dr. H. A. Smith, had used it some, and thought, no doubt with use, a person could acquire the facility to use it very well. He found some difficulty, however, in using it, losing control of the plugger on raising the piston to strike a blow.

The exhibitor thought there was no difficulty in regard to controlling the plugger, one could easily strike a light or heavy blow as he wished.

Dr. Scroggs had told him he would quit filling teeth if it was not for that instrument. Some one thought the sound it made was objectionable. Dr. Keely thought this could easily be remedied by having it strike against a little washer made of leather and nicely fitted in to deaden the sound.

Dr. Watt was much pleased with the instrument in some respects, but objected to it on the ground of its striking what might be called a dead blow. The Dr. gave two or three illustrations to show the difference between a dead blow and what might be termed a live blow. It was much easier to drive a nail in a springing weatherboard with a small tack hammer than with a heavy hammer.

He objected to using a heavy mallet from this same principle. In proportion as we deaden the stroke we increase in the same proportion the pressure on the periosteum.

The insertion of leather in the mallet to deaden the sound which some of his brethren had suggested, and the use of a heavier mallet to produce the required force, was unphilosophical. If two balls of equal weight, one of marble the other of rubber, were dropped on a marble floor from the same height, the marble ball though much the smaller, would produce the greatest effect on the point struck and rebound more instantaneously, because it struck a *live* blow; so, if a pane of glass were suspended by a string and you were to strike it with your fist, or throw a stone against it, it would be dashed to pieces; but fire a pistol ball through it and it makes a clean nice hole. Here is a *live* force, a condensed force. This is just what we want in condensing the gold. It is not a bit of difference if the mallet does make a noise; the patient will think something is wrong if it does not. And it is in fact a guide to the operator and assistant; they will know whether the right stroke is made or not. If then we want this live stroke instead of the heavy stroke as suggested, we must use the light mallet and make quick, free strokes. A light mallet with long handle and quick stroke, is the one that will give the most force on the surface of the gold, and the least concussion to the periosteum.

### DENTAL SPECIALTIES—THEIR ADVANTAGES AND DISADVANTAGES.

Dr. Watt supposed the question was whether there was any thing gained by sub-dividing Dental practice; whether or not, our profession would be elevated by further subdivision. We have some men who have turned almost their exclusive attention to mechanical Dentistry; such men generally excel in mechanical Dentistry the practitioner who practices the whole range of Dentistry. There are others who fill teeth and do nothing else, and are more likely to excel in this than others who put three-fourths of their time in the laboratory, and who fill teeth only when they can not help it. Others devote their time to diseases—the constitution of the patient likely to result in disease, etc.

We find these sub-divisions—persons devoting themselves to these specialties—in such cities as Cincinnati, New York and Chicago. My opinion is that it will become the practice in almost every city, for Dentists to practice in a special department to the exclusion of all other departments. He had been driven in that direction himself, because he had become physically disabled, and could not practice the whole range of the profession. He had paid more attention to diagnosis and what might be called the surgery of the mouth and jaws, and though not in a condition for active mental, effort, yet from the mere fact of his having turned his attention in that direction he had made considerable progress.

Every Journal, medical or Dental that he found, he examined to find everything that would throw light in that direction, and in conversation he had obtained much information in this way, gathering up every thing that had a bearing on the specialty he endeavored to practice. This turning his attention in one direction enabled him to take hold of the subject in a way he never could before, when his attention was divided over the whole range of subjects belonging to the profession.

Again, in regard to the disadvantages; he knew very well, if he continued in his present course, he would fall behind in mechanical Dentistry, and operative Dentistry proper. But the question was, not what was good for one man, but for the *profession.*

He was opposed to the old theory that if a person had a genius in one direction, it must be corrected by exercising his faculties in that for which he had but little taste. If every man were an Alexander Selkirk, it might be best to do this; but man was made for society. If he fell behind in operative or mechanical Dentistry what matter, provided his brethren pushed on these departments to a greater degree, just as he should the department in which he was trying to labor.

He thought by each one taking that department for which he was best fitted by taste or talent, or surrounding circumstances, the whole profession would make greater progress.

He confessed that he was in favor of Dental Specialties in the profession, theoretically and practically.

Dr. J. B. Beauman, of Columbus, O.—For the last two years he has been confined almost to the department of Operative Dentistry.

The longer he turned his attention in that direction, the more he saw the necessity of specialties in practice. He was well satisfied in his own mind, that the time would come when the profession must be divided up into specialties.

The great difficulty that stood in the way with many, was "will I be enabled to maintain myself, and make a living if I direct my attention to one branch of the trade? Will I succeed"? That is the great trouble. But he held that it is the duty of every one in the profession, who sees the subject in this light to advocate it until the profession is brought up to a standard, where we can go to work in earnest in specialties. Dr. White would tell them that he can carry on his business with greater success, by having his hands employed, one in one particular branch of his business, and another in another branch, than if all worked promiscuously, and that each one, by being confined to a particular branch, became much more proficient in that, than if he worked in all the different branches of the business. The same was true in any large manufactory, each one could perform his part of the work better than any other man in the manufactory.

It seemed clear to his mind that such a course was demanded in the profession of Dentistry, and not only this, but he held that community demands it at our hands. He was well convinced that in pursuing but one branch, they would make far greater progress, than when pursuing all combined. If a man turns his attention to one line of business, he would be much surer of success.

Dr. C. W. Spalding, of St. Louis.—Said he supposed no man would practice the art of Dentistry many years, especially if he attempted to practice both the mechanical and operative departments, without finding it a great inconvenience to attempt to practice both these departments conjointly.

Certainly, the mechanical department in itself, presented a wide range—an endless number and variety in the cases presented—and was certainly a sufficient field for all the genius and mechanical ability which any one man may be supposed to possess. It has been his opinion for years, that this necessity for a division of the departments of Dentistry in practice, would force itself on the profession.

He had altogether abandoned mechanical Dentistry, because he had a taste for operative Dentistry. By degrees he had lessened his practice in mechanical Dentistry, until it amounted to nothing. He believed that this sub-division of the profession would be the inevitable course in large cities. The sooner it was brought about the better, he thought, for the profession. The operator would acquire greater skill, by having his attention turned to that one department.

The executive ability or skill—the manipulative ability that was indispensable, would be destroyed by turning his attention to some other occupation. The more practice he had in this branch, of course the greater ease and facility he would acquire in doing this kind of work, and with more ease to the patient.

He thought it did not need much discussion. It must seem better, he thought, to almost every one. He had certainly been long convinced of it, and thought it the duty of every one to urge it to be put in practice, and try to make it general.

Dr. J. Taft.—Said he supposed it would be a better state of affairs if it would be adopted at once, but the question arises, "is it practicable?" Many men in the profession are compelled under present circumstances to practice everything. But while that might be the case under some circumstances, he thought it need not be the case to as great an extent as was supposed.

He was well convinced that very much more could be done than is done in this way, if the matter could be urged and pressed upon the attention of the profession.

He thought in every city, in every village or town, where two or more Dentists practice, there could be an arrangement of that kind made, and that they should seek to make such arrangements. In every town, as it now is, where there are three or four Dentists they are driving ahead at all they can lay hands on, in all departments of the profession—operative Dentistry, mechanical Dentistry, the treatment of diseases—complicated and simple—treating irregularities, and taking hold of everything within reach. If, where there are two, or three, or four Dentists in the same vicinity, they would make an arrangement for each one to pursue that for which he has the most aptitude—the greatest liking, then all would be harmonious. But where men endeavor to do everything, there is a competition that is many times injurious, and operates against the profession, whereas, if, for example, in a village where there are but two Dentists, they would make this division, one taking one branch and the other another branch, and each co-operate with the other, and help build each other up, they would help create in community, that respect and regard which ought to be manifested towards them, and towards the profession in which they are engaged. But instead of this, there is now in many places, a mean contemptible competition that produces almost—yes, sometimes quite—disgust in public opinion. By the kind of arrangement referred to, all this could be cleared away. This ungenerous competition would be destroyed, and each one would occupy a far better position, by taking each a specialty, which they would develop to a much higher state of perfection. There is no doubt if they would thus work together, and be harmonious in their aims and objects, they would create in community a sentiment—a feeling of respect—which never existed before.

This plan is feasible if men will but take hold of it, where there are but two or three Dentists in a place.

It is working well in cities, naturally working itself out. What he urged, in smaller places where it does not seem to separate itself thus, was an effort to make that separation, so that all would go on harmoniously instead of this kind of competition, so common as to be almost universal, tending to drag the whole matter down and make the profession an object of contempt in the minds of the people. It would be an important matter if no more than this was accomplished. These things had forced themselves on his mind for a long time.

True, there were noble exceptions, but in many places it seemed that Dentists took pride in condemning the practice of each other all they could.

The question was asked Dr. Taft, how many sub-divisions he would make?

The Dr. replied that it would depend upon circumstances. In this city and other large cities, some turned their whole attention to mechanical Dentistry, making artificial teeth, &c. Others did nothing but fill teeth.

As an illustration, in their office, one did not do much else than attend to the department of mechanical Dentistry, another, nothing scarcely but fill teeth, and for sometime he had treated no cases of irregularity, but had turned that over to another person in the office, who had done it far better than he could or ever did, with all the different branches mixed up together. His operations were now confined to filling; he had centered down to the one work.

Where there were but two Dentists in a place, they could not carry their sub-division as far as in cities. One might perhaps treat diseases and fill teeth, and the other attend to the mechanical department, and by helping build up a business for each other, would raise the profession in the estimation of the people around about them.

Dr. Talbert remarked, that it was a well-known fact, that men acquired skill and excellence in a separate department, that they never could acquire if they practiced all. He had investigated the subject, and made the discovery that these are the men that make those improvements which are of importance to the public. We never saw a man yet that practiced a specialty, who was not a liberal one. He might cite, as evidence of the fact, that those improvements are most readily given to the public by such men.

Dr. Berry, said, he agreed with nearly all the remarks made on the subject, but there were difficulties in little towns, where there were but two practitioners. So far as the good of the profession was concerned, he had no doubt that the course proposed was better, than for one person to practice in all departments.

He was very much pleased at Dr. Watt having turned his attention to the special department he was pursuing. He had turned over to the Dr. some bad cases for treatment of the mouth, that he himself did not wish to bother with. There is but little chance to give proper attention to such cases, in an office where one has forty other things to attend to. Perhaps some one is waiting to have something done in the department of operative or mechanical Dentistry, and the practice may not justify the keeping of an assistant at twenty dollars per week—a thousand and forty dollars per year. It would be better to let such cases slide once in a while. There are, however, so many cases where we are forced into doing that which we would prefer others to do.

Dr. Taft.—In the arrangement he had spoken of, he would have it understood, that it was simply making a trade, simply turning over work from one to the other. The man to whom you turned over work in his line, compensating you by turning over to you work in your department. Another thing, as each one would be confined to one thing, that part would be much better attended to, than when you attempted two or three things at a time.

Thus, by pursuing this course, by study, and practice, and experiment, you will improve so, that your operations will be brought to a greater degree of perfection, and secure you more patronage in your special department, which in addition to the work turned over to you, by the person with whom you have this arrangement, it seems to me, will more than compensate you for cutting off one department of the business.

Dr. Berry wished some plan could be devised to enlighten the people to attend to their teeth earlier, and to impress them with the value of filling them, instead of letting them decay.

Dr. B. F. Rosson, said in his town his neighbor Dentist gave exhibitions with laughing gas in connection with his other business, and enquired if he should encourage him in this undertaking. (Laughter.)

Dr. J. B. Beauman, asked how many would go to the general practitioner of medicine, to get a difficult surgical operation performed. It struck him that very few would do so, because surgery and the general practice of medicine have been divided, and it is naturally expected that the Surgeon becomes more expert in that branch, than the general practitioner. This is supposed because he makes it a specialty, and any one that wants anything in that specialty goes to one who makes it such. There is hardly a man in the house, if he wants his teeth filled, that would go to the operator who tended to all parts of the profession—mechanical, operative and the entire work of the Dentist, but would seek a man who made that a specialty, because they expected to get a far better operation performed by such a one. He wants his teeth filled well and saved. And it will be the same way with persons in a community; they will learn where to go as soon as the profession is divided, and specialties made of it. Many thoughts might be suggested on the subject, but it seemed apparent to him that every one must see at a glance, the necessity of the profession pursuing specialties.

Dr. C. M. Kelsey, thought in cities like this it was quite practicable, and he was in favor of the plan where it would work. Where specialties were practiced, persons certainly became more efficient and did better work, but it was certainly impracticable in country towns. Take a town of the size of his, where the population did not exceed five thousand. If you would try to make an arrangement of this kind, you would see how it would turn out. There would be a disagreement as to who should take the operative, and who the mechanical department, &c. Besides there are families who have put themselves under our care, and expect us to do all their work for them, whether mechanical or operative, and if you would turn them over to another, to get a part of the work done, they would think it was something not very profitable to you, and you wanted to get rid of it, and some would take offense, and you would thereby lose their patronage altogether. Though where practicable, such practice was best, but he thought it would not give satisfaction in smaller towns.

Dr. Taft said Dr. Kelsey seemed to look on this matter as wholly impracticable in towns. Perhaps it would be so in many places at present. Persons must not think that it can be accomplished right at once, but if everybody would go to work, and endeavor to bring it about, it could be done after a while. There were difficulties connected with everything in life. It would not do to make mountains of difficulties that were but mole-hills. If we would just begin to climb them, we would surmount them much easier than we expected.

Dr. Rosson, thought we would find, if we sent a turkey, a crow would be sent us in return.

Dr. Berry believed there were many such men where the arrangement would be perfectly impracticable.

Dr. Watt, thought we should not be discouraged at some little difficulties. As he looked around over the assembly there were some bachelors to be seen. He did not know how to account for the fact they were not married. He supposed some were afraid to go to see the girl they liked, for fear some other fellow might go to see her too. Some were afraid to adopt special things, for fear his friend might want that specialty too. For his part, if he wanted a girl he would not stay away because some other fellow wanted her. He would urge the adoption of specialties wherever practicable.

He knew there would be difficulties in some places, and in some places they could not be introduced. Marriage was the natural state of mankind, but there had always been some bachelors. So, though specialties was the natural tendency of the profession it would not be carried out in all places.

If, for instance, Mount Vernon, Dr. Kelsey's town, thought he was better calculated to work in the department of operative Dentistry, than any other department, they would go to him when wanting anything in that department, and another person intending to practice the same specialty would hardly locate there, but soon mechanical work would run in and require the services of one in that direction. It might take some time to bring this about, but there would naturally be this tendency.

Dr. G. W. Keely, said his mind was impressed with the importance of specialties.

In the winter of 1842–3, which he spent in New Orleans, Dr. Palmer, of that city, made a specialty of filling teeth. In conversation with prominent men, physicians, lawyers, &c., he found that a great majority went to Dr. Palmer to get their work done, because he did nothing else but fill teeth. Dr. Keely felt safe in saying if he could get the prices operative Dentists get in cities, he could make more money in the place he lived, where there are but three thousand inhabitants, than he did now, and could do better work for his patients. He had recently filled a bicuspid tooth, which occupied an hour and a half to perform the work. The patient counted the taps of the mallet given—he did not know whether such a thing had ever been done before—

and he was surprised at the large number. He had given 2782 taps of the mallet in filling the tooth. He was more than ever convinced of the value of the mallet. He did not pretend to say good filling could not be done without the mallet. Dr. M. Rogers, years ago, had put in fillings by hand pressure, that had done well; he knew some men had a better sleight of hand in doing it thus, than others. He was accused of having mallet on the brain, but he thought when one got sufficient practice with the mallet, to learn its importance, they would have mallet on the brain too, and his patients would have it also. (Responses of "that's so.")

He was convinced of the necessity of making certain parts of the work a specialty. Suppose a person desiring to have an operation performed on an eye, should come here to Cincinnati for the purpose, he would certainly go to some one who makes the treatment of the eye a specialty.

### DISCUSSION ON OPERATIVE DENTISTRY.

Dr. Berry mentioned a case of a patient with the second molar nerve destroyed; treated, filled with amalgam, three years elapsed before pain. In removing the filling, found living nerve in the anterior fang; the other canal was open and clear; no indication of suppuration.

Dr. Taft.—In reference to the treatment of exposed pulps, think that in many instances it is proper and even best, to let the pulp tissue remain in the canals of the roots, after that portion in the chamber, in the crown of the tooth, has been removed. That at the point of excision cicatrization may be induced, and when this can be accomplished, and there are existing no unfavorable conditions—neuraligic, irritable or inflammatory, it is best to permit the pulp-tissue to remain in the roots; protecting them at their exposed points, and filling the vacated chamber and the decayed cavity. Dr. Garkey, of Memphis, Tenn., has employed this practice for a considerable length of time, and with very marked success. If the exposed points are well protected from variations of temperature, and there is no change of the material that is placed in contact with them, and there neither should be a vacuum nor impingement, and all will be well.

No plan can be devised that will operate alike well in all cases. This is only sought by the superficial men in the profession who are too indolent to devote time or effort to the development of a special treatment, for special cases. For intervening substance make use of Hill's stopping. No space should be left, and yet pressure on the pulp must be avoided.

Dr. Berry asked whether the treatment of filling the fang with cotton and creosote, does not answer the requirements?

Dr. Taft thinks this sufficient as it is only necessary to prevent the admission of moisture to the canal.

Dr. Spalding—the essential point is to close the foramin. The infiltration of moisture through the wall is an unsettled point. He did not consider it essential, that the cotton should remain longer than a few moments, to form carbolat of albumen, as the chemical effect is produced in a few moments. Always endeavor to render the whole fang impervious to any fluid. He had not lost more than two per cent. of these cases. He objected to cotton, because of quacks.

Dr. Taft always uses small pludgets of cotton with creosote. Uses cotton because not so liable to extend through the foramin.

Dr. Berry would not reject the use of cotton because of quacks, but would practice any method adapted to the case in hand.

Dr. Morgan spoke in reference to the use of carbolic acid as a preservative against decomposition. A subject injected, will be preserved for an indefinite length of time.

[The foregoing on operative Dentistry is from notes taken by the Secretary.]

Dr. C. M. Kelsey remarked that he practiced filling the cavities with cotton, saturated with creosote; perhaps it was as good as any other filling; thought it less liable to pass through the foramin. He wished to say a word in reference to what some gentleman said last year with regard to the practice of extracting the six year old molars. The gentleman advocated the propriety of extracting it, when but slightly decayed. This was contrary to his practice and teaching; he endeavored to save it as long as practicable, and instructed students and patients when they first decay to have them filled. He thought there was an error in extracting these teeth too soon, he would let them remain as long as there was any attachment, unless other teeth took a wrong direction. The reason he preferred not to extract the temporary teeth, was that the child at that age—nine, or ten, or twelve years—was tender and it was a pretty severe operation, and would cause the child to be forever set against going to a Dentist. He would have particular care to save these molar teeth. If the mode of others was different, he could not help it, and supposed he might be considered an old fogy, but he really considered the practice reprehensible and never adopted it.

Dr. Shadoan, remarked that he agreed in a great measure with what had been said.

The first thing that attracted his attention, about which he desired to speak, was a remark of Dr. Taft, with regard to destroying the pulp. He had quoted Dr. Garkey, but there was one thing he failed to mention; that is in destroying the pulp with anything, he leaves it in just long enough to devitalize or destroy a part of it; then make an application of cotton or lint, saturated with creosote, to form a cicatrix or heal the nerve. If let remain two, three or four days, we will find a little dark speck on the saturated lint, opposite the roots, showing that the healing process had commenced.

In regard to filling with any material after the nerve was removed, he differed with Dr. Spalding, who did not want any cotton left in the cavity at all.

Dr. Shadoan did not know that he ever failed. His method was to place a small portion of cotton, lint, or a few fibers of flax, saturated with creosote. Flax thread or silk thread would do—he preferred flax—and forced it up as far as he could into the fang.

He next spoke of the danger—in case of a slight change of the drill—of the instrument running through into the periosteum of the tooth, and gave some instances where this had occured. He said it could be determined just how far to force the cotton or gold. It could be determined by measurement. For instance, take the instrument, an excavator, and when it gets through, let it catch over the wall, then measure; then change and feel the other way. Practice in one or two cases was generally sufficient to learn how to measure the depth. The instrument must be allowed to go as far as it would, making allowance for thickness; should not press the instrument too hard lest it be forced through.

He never filled more than the fangs of the teeth with os-artificial; filled the cavity with os-artificial, which was better because it forms a more solid base, and filled the remainder with gold.

One reason for using os-artificial was, that it was a non-conductor, when a chill only goes to the os-artificial, and is not conveyed into the tooth. We often find that a change of temperature produces periostitus; and many teeth are lost when they are well filled.

A remark or two in reference to the six year old molar teeth; he considered these of as much importance as any in the mouth; in his opinion, they were made of better material than any other in the mouth. If we have found them decaying, it is not owing to faulty material, but the mode of living.

He thought they were formed of better material, and were placed in the most important part of the mouth, and performed the most important office of any other, in preserving at that particular time, the symmetry of the jaws and face, and should therefore be retained on that account.

Dr. Talbcrt would like to say that he preserves so far as he can, the deciduous teeth; thinks it an important point, in order to preserve the arch of the mouth. He had even sent parents with children home, whom they had brought to him, to have their teeth extracted. He advised them to leave them in the mouth; sometimes he found that they had gone to other Dentists who had them taken out. Some of his patients —children who had been brought to him by their mothers, he had sent home two or three times, advising them to leave them in as long as possible. He would have it distinctly understood, when he found the first molar teeth in the mouth well developed, and having abundance of room, he would not take them out, but when crowded and having two or three cavities he would extract. Dr. Talbert then gave the history of some cases that he had treated, as illustrations of the succsss he had obtained by pursuing this course.

Dr. Morgan thought there was a large number of cases in which it was impracticable to save the first molars, especially among the children of the more wealthy, whose habits were more luxurious. It was difficult to lay down a rule to which there was not a number of exceptions—all general rules have. It was a nice question to determine; of course it was modified by surrounding circumstances. It seemed we could come to no general rule. If those teeth were beyond the reach of our skill to save, until the patient approaches or arrives at maturity, he had believed it best to extract them at an early day. But the period when to extract was difficult to determine. As a general rule, he would say retain them as long as they produced no pain, or could be used—were serviceable. It was certainly desirable for the child at this age to have their teeth to masticate with, while the formative processes were going on. There was no period when it was more necessary to have good grinding apparatus, than just at this age—from seven to fifteen.

He considered the first [molars the most valuable, except the canine teeth perhaps, in the mouth.

REMARKS ON DR. SPALDING'S ESSAY.

Dr. S. S. White—It is not without feeling that it looks like presumption in me, that I attempt to say anything after the very able essay that has been read in your hearing. The first thought that I wish to give expression to, is in relation to the culmination of the idéas presented in the Doctor's essay. If the theory be correct in regard to the dentine, it would seem that the remedy should have been found in congealing the parts by rhigoline spray, to prevent that vibratory motion. This has been tested pretty thoroughly in the East, upon those having quite sensative teeth, without injury to the pulp.

In regard to another matter: I wish to offer some remarks to instruct Dentists how to take care of their health.

I don't speak in public often enough to keep the tether of my thoughts. I will endeavor, however, to make some observations on this subject. Some of my old friends know that some ten years ago, I presented the appearance of a very delicate man, likely to have a short life. That condition opened my eyes more particularly to the laws of health.

It was in studying some of the authors, quoted here to-night, that I was led to pursue the plan I did.

I wish to give you some of my experience, which I hope may be of immediate service to you. We recognize the higher power of nerve force, as the highest we know anything of in our organization—that which enables us to think. I have proven in my own case, the value of the principles stated, having brought myself from that of an emaciated pale faced person, weighing but one hundred and twenty-one pounds, to that of a person weighing one hundred and seventy one pounds, with a full face and florid complexion, in which there is no alcoholic or other stimulant. I accept it as a truth that the most immediate attainable power for the conversion into red blood globules, and converting it into gray matter, is the direct rays of the sun. I have put it in practice and tested it. We know that it is so in vegetable, flower or fruit growth. The fact that vegetables deprived of light do not develope themselves fully is well known to all. But where they are furnished with abundance of light, but deprived of direct rays of the sun, the vegetation does not develope itself to the same intensity—the higher qualities of the fruit is not developed, although exposed fully to the northern light; they must have the influence of the direct rays of the sun also.

To make this applicable; in looking around on the Dentists here, I find that they partake of the general character of Dentists; whatever their constitution, they are pale, owing to their inactive in-door occupation, keeping themselves shut up six days in the week out of the sun.

If I were their medical adviser, I would advise them to take one or two hours in the middle of the day to expose themselves to the direct rays of the sun.

The habit will be easily acquired, and will become a luxury, even when perspiration is freely developed in the sun, in the hottest days of the year. When in health it is the best way to retain it, and if out of health, the best way to regain it.

I have found when overworked by brain work, as I have sometimes been, that the readiest means to recover my health, was by exposing myself thus to the direct rays of the sun.

When the Doctor spoke of sleep as a means of restoring the gray matter, it reminded me of the fact, that when thus exposed to the direct rays of the sun, I needed but six hours sleep, when I otherwise needed eight hours.

Dr. Warder, who is an experienced horticulturist, in speaking on some of the points introduced by Dr White, said that tropical plants, which of course existed under the immediate or more direct rays of the sun, than we had here, could be grown by artificial means, as in our green houses, we could raise a sufficient amount of heat, but they never thrived so well or produced the same result as under a vertical sun. He fully believed the Doctor's remarks with regard to health; he did not know about his rosy cheeks, but he had a figure he was not ashamed of; one much superior to the one he carried about with him when he lived in Cincinnati. He had changed his appearance since he had become a Farmer and had left off being a Doctor; he was not shut up as he was then.

It is well known that the fruits well exposed to the light and sun, are universally better developed in size, infinitely better in flavor, and much more highly colored than those that are not thus exposed.

Dr. Talbert wished to add his testimony to the fact, that by reducing the temperature by ether spray, the dentine would have no sensitiveness whatever in it, and one can then work and cut away as he pleases.

Dr. C. M. Kelsey.—There seemed one thing not very clear to his mind, in relation to the dentine. The question in his mind was, what gave the sensibility or sensitiveness to the the dentine, if the nerve fibril did not pass into the dentine.

Dr. Spalding—No nerve fibrils had been found in dentine. There had been found what was supposed to be such, but they have not been traced further through than the outer membrane of the soft pulp.

The nerve fibrils have never been traced through that membrane or into it; some have said they did so, but this has never been corroborated by others. They have never been traced into but all terminate underneath it. None have ever been found perforating or passing through, and I cannot imagine how they get into the tubule of the dentine, when they have never been known or seen to pass through. This is a question I am unable to solve, and am forced to the conclusion, that as they have never been found to exist in the dentine, they do not exist there. The question then is how can we account for the sensibility, without their presence. The theory I have given, satisfactorily accounts for it.

If we touch the finger to any surface, though the nerves do not come immediately in contact with that surface—though there is no contact except of the outer substance—the motion is communicated to the nerves and through them to the brain.

### DISCUSSION ON THE QUESTION.

*Can anything be accomplished for the arrest of Dental caries, either by general or local treatment, otherwise than by filling?*

Dr. Morgan hoped to hear especially from the faculty on that point.

Dr. Taft supposed the question would refer to both the predisposing and the exciting causes; everything, either of a general or local character.

He supposed all were familiar with the fact, that the more perfect the condition of the patient, in any case, the less liability there was to decay in the teeth, and the less rapid the decay. The question he supposed to be simply this: To know what can be done, so far as predisposing causes exist, to clear them away.

A great deal could be accomplished by thoroughly understanding the patient's condition, by making a thorough diagnosis—to know what are the predisposing causes, and the exciting causes; to find whether it was enfeebled vitality, or whether any special virus, syphilitic or any other poison was present; or the production of any vicious secretions that may facilitate the progress of decay. He supposed that anything that would improve the general health, would be the means of arresting decay.

How much, or in what ways this can be done, of course is for us yet to determine, but facts are cropping out, that should lead us to make inquiries and observations in this respect.

He referred to the case spoken of by Dr. Spalding, in which, by a certain treatment the softening of the teeth was arrested; and referred also to an instance in which the teeth of a lady became very much softened by some disease, in a comparatively short period of time, so that they could be cut away as easily as a piece of wood, but by a certain course of treatment they were hardened or solidified in a short period of time, and made much more dense than before. Things of that kind were occasionally coming to notice, which were very significant, and ought, when they occur, to be traced up fully and investigated, so as to learn all we can about them. Though we may not understand all about them, we should pursue the matter and endeavor to *learn* all possible about them.

He supposed anything that would give increased tone and vigor to the system and improve the health, would of course tend to arrest caries, and anything that will *prevent* decay will tend to *arrest* it during its progress. Many times we find the teeth decaying, and pay but little attention to the condition of the patient or to those things which cause the decay. We fill the cavity and let the case go without any special notice, or without trying to find out what are the causes of decay, or without recommending, or introducing such a course as shall remedy it, or prevent an occurrence of the difficulty again. He thought this a great fault in the profession—they find a cavity in a tooth, clean it out and fill it well and think they have done their duty.

He thought they should try to ascertain whether there were any systemic influences, and what local causes for the decay; to find out if possible what the sources were, from whence they emanated; whether from vicious secretions of the mouth, or from secretions in the alimentary canal, or from the decomposition of food, or other sources. When we find out the sources or agents that produce the effect, we will best know how to control them. It is not enough, however, that we have ascertained the character of the agent or the source from which it comes, but we should endeavor to find out how best to conquer that enemy. It is not enough to know that an enemy is at such a point, and intrenched so and thus. But in order to attack him successfully, we must know how best to approach him, and how to neutralize the advantages of his position, &c. Just so here, we may have a very good understanding of the agents that produce decay, but we must know what to do to meet the difficulties, and counteract these influences, and how to clear them away or neutralize them. When there is an increased vigor, or the general condition of the patient is improved, there is generally a corresponding arrest of decay, a solidification of the dentine. We should study all the conditions in connection with such cases, and if we study them closely, we will learn much.

It is an important subject, one that should be carefully considered.

In some instances we dress the teeth, file away the decayed portions, thinking to arrest decay, but the decay persists; at other times the teeth without this will be found years afterward to have ceased decaying, and the surface to have become more dense than before—there has been an eburnation of dentine, almost as dense as enamel. The improvement of the general health is one great remedy; care of the teeth themselves—keeping them in the best possible condition, giving them proper exercise, keeping the gums in a healthy condition; the mouth in good state, will tend much, not only to prevent decay, but to arrest it wherever commenced.

Dr. Watling desired to ask a question: The teeth of a patient of his, a lad ten or twelve years of age, just recovering from an attack of typhoid fever, were so affected that the enamel was so soft as to easily scrape off. After scraping through the soft enamel to the dentine, it was hard and solid, and the teeth were not sensitive. He would like to know what course to pursue in the case?

Dr. Taft said it was an important case, one that would require a close observation, a thorough diagnosis to ascertain what the cause of the trouble was; whether it originated from systemic causes, or whether by the action of local agents. He supposed it likely resulted from vicious secretions of the mouth.

He would determine, if possible, the nature of the secretions of the mouth, whether acid or not in character, and pursue such a course as would change the secretions by general treatment. It involved the whole subject of systemic treatment. Dentists should understand all this.

So far as local treatment is concerned, he would scrape off the soft enamel down to the dentine, and give it as smooth and solid a surface as possible. Would use as a mouth wash some alkali, prepared chalk, or lime water, also phosphates.

The general treatment must be modified of course by the condition of the patient, would use such medication, or hygienic treatment as to bring about a healthy condition of the patient.

Dr. Watling remarked that the patient had been sick, but that the secretions of the mouth were now in a perfectly healthy condition.

Dr. McCullom believed the case referred to by Dr. Spalding, was caused by an excess of alkali being taken into the system. The appearance of the teeth improved rapidly after eating acid fruits.

Dr. Shadoan, had met with cases, not unfrequently, of children, twelve, fourteen, or sixteen years of age, whose teeth presented a greenish appearance, more especially the front teeth, around the surface next to the gum, the enamel scales off, &c. What caused it, was an important consideration. It was his opinion, it was produced from the acid secretions in the mouth. Sometimes it extended to the cuspid and bicuspid teeth, but generally only to the incisors. As it is principally on the incisor teeth, he thought it was caused by the vitiated condition, or acid condition of the mucus; that class of people generally sleep with the mouth open, and the mucus accumulates and dries on that part of the mouth.

To correct that, in the first place the acid secretions must be corrected, which may be done as Dr. Taft remarked, by using prepared chalk, lime water, or anything that would change these secretions of the mouth. Then in case the patient sleeps with his mouth open, he would recommend the mouth to be kept closed and get in that way of sleeping. He would also have them wash the teeth well, especially at night before going to bed. This he thought would go far to prevent these difficulties and caries in the teeth. The sooner the soft parts are taken off the teeth the better. These deposits should be removed and the teeth put in better condition, and washed and kept clean, when friction of the lips assist in keeping them clean. This is a matter that is not understood as it should be, but we all ought to understand the nature of the secretions of the mouth and of the system generally, whether they come to the surface or not, and what will change the condition of these secretions.

